# Efficacy and Safety of Prolonged Adjuvant Temozolomide Treatment in Glioblastoma: Prospective Study of 81 Patients Undergoing up to 101 Cycles of Treatment

**DOI:** 10.3390/brainsci15050428

**Published:** 2025-04-23

**Authors:** Giulio Bonomo, Francesco Certo, Erica Grasso, Giuseppa Fiumanò, Davide Barbagallo, Rosario Caltabiano, Giuseppe Broggi, Gaetano Magro, Andrea Maugeri, Antonella Agodi, Fiorenza Latteri, Hector Sotoparra, Giovanni Buscema, Corrado Spatola, Alessandro Pluchino, Giuseppe M. V. Barbagallo

**Affiliations:** 1 Department of Neurological Surgery, Policlinico “G. Rodolico-S. Marco” University Hospital, 95121 Catania, Italy; dott.giuliobonomo@gmail.com (G.B.); cicciocerto@yahoo.it (F.C.); egrasso360@gmail.com (E.G.); 2 Department of Medical and Surgical Sciences and Advanced Technologies “GF Ingrassia”, University of Catania, 95123 Catania, Italy; rosario.caltabiano@unict.it (R.C.); giuseppe.broggi@gmail.com (G.B.); g.magro@unict.it (G.M.); andrea.maugeri@unict.it (A.M.); antonella.agodi@unict.it (A.A.); cor_spatola@hotmail.com (C.S.); 3 Interdisciplinary Research Center on Brain Tumors Diagnosis and Treatment, University of Catania, 95123 Catania, Italy; gfiuman@sirm.org (G.F.); dbarbaga@unict.it (D.B.); f.latteri@policlinico.unict.it (F.L.); hsotoparra@policlinico.unict.it (H.S.); 4 Division of Radiology, Section of Neuroradiology, Policlinico “G. Rodolico-S. Marco” University Hospital, 95123 Catania, Italy; 5 Department of Biomedical and Biotechnological Sciences, Section of Biology and Genetics, Policlinico “G. Rodolico-S. Marco” University Hospital, 95121 Catania, Italy; 6 Division of Anatomic Pathology, Policlinico “G. Rodolico-S. Marco” University Hospital, 95123 Catania, Italy; 7 Division of Medical Oncology, Policlinico “G. Rodolico-S. Marco” University Hospital, 95123 Catania, Italy; 8 Department of Anaesthesiology, Policlinico “G. Rodolico-S. Marco” University Hospital, 95123 Catania, Italy; buscemagiovanni@gmail.com; 9 Radiation Oncology Unit, Policlinico “G. Rodolico-S. Marco” University Hospital, 95123 Catania, Italy; 10 Department of Physics and Astronomy “Ettore Majorana”, University of Catania, 95123 Catania, Italy; alessandro.pluchino@ct.infn.it

**Keywords:** extended, glioblastoma, long-term, STUPP, temozolomide

## Abstract

**Background:** Although several studies investigated the efficacy of long-term adjuvant temozolomide (TMZ) therapy in glioblastomas (GBs), no univocal data are currently available, and this topic remains controversial. The present study on our ongoing experience aims to assess whether the extended STUPP protocol confers prognostic benefits with acceptable safety. **Methods:** From 2004 to 2018, 81 patients with a new diagnosis of GB according to the World Health Organization (WHO) 2021 classification, treated with gross total resection (GTR) or subtotal resection (STR), were enrolled. Patients were divided into Group A (long-term TMZ; N = 40) and Group B (standard STUPP protocol; N = 41). **Results:** In the extended STUPP group, compared with the standard STUPP group, progression-free survival (PFS) and overall survival (OS) were significantly improved (PFS: 27.8 vs. 7.5 months, *p* = 0.00001; OS: 35.9 vs. 11.3 months, *p* = 0.0001). To mitigate a potential survival bias, we focused on those in Group B who completed the recommended six cycles. Patients in Group A demonstrated a prolonged OS compared to Group B (27 vs. 10 months, *p* < 0.001). Similar findings were observed in a focused analysis of patients who had achieved a minimum survival of 12 months (27 vs. 15 months, *p* < 0.001) or 18 months (34 vs. 24 months, *p* = 0.044). **Conclusions:** Our analysis demonstrates a PFS and OS advantage with extended STUPP and suggests that young patients without corpus callosum invasion, with methylguanine-DNA methyltransferase (MGMT) promoter methylation, and treated with GTR are the best candidates. No significant safety difference emerged between extended and standard TMZ treatment.

## 1. Introduction

Based on the 2005 Phase 3 Trial by Stupp et al., the standard of treatment for patients with a first diagnosis of glioblastoma (GB) is safe maximal surgical resection followed by radiation therapy plus concomitant temozolomide (TMZ) chemotherapy, then followed by six cycles of adjuvant TMZ [1].

There is no consensus in the literature regarding the optimal duration of adjuvant chemotherapy with TMZ in GB, and some authors, even the ones of a recent combined analysis, advise against long-term TMZ because the analysis of the data did not demonstrate a survival advantage in terms of overall survival (OS) and progression-free survival (PFS). However, these studies often lack an analysis of GB according to the current World Health Organization (WHO) 2021 classification, considering different extents of resection (EORs) including biopsies, and do not identify subgroups of patients who might benefit the most from extending the STUPP protocol [2]. The present study aims to assess whether the extended STUPP protocol confers prognostic benefits with acceptable safety.

## 2. Materials and Methods

Patients prospectively enrolled from 2004 to 2018 for GB surgery were analyzed. Inclusion criteria were primary diagnosis of GB (also confirmed according to 2021 WHO classification after further biomolecular review); ≥18 years old; confirmed gross total (GTR) or subtotal resection (STR); postoperative concomitant radiotherapy and chemotherapy according to STUPP protocol; and adequate hematologic, renal, and liver function [3].

Data collected for each patient included the following: age, sex, Karnofsky Performance Status (KPS) preoperatively and six months postoperatively, tumor location, ependyma and/or corpus callosum invasion, preoperative tumor volume, EOR, postoperative tumor volume, OS, PFS, methylguanine-DNA methyltransferase (MGMT) methylation status, radiation dose, number of TMZ cycles, and chemotherapy-related complications.

Chemotherapy-related complications were classified according to Common Terminology Criteria for Adverse Events (CTCAE) [4].

All patients underwent postoperative magnetic resonance imaging (MRI) within 48 h. Tumor volumes and EOR were assessed with manual and semiautomatic segmentation (StealthVizTM, Medtronic, and Advantage Workstation VolumeShare 5, GE HealthCare) by two expert neurosurgeons in neuro-oncology.

The decision to prolong TMZ therapy beyond 6 months was based on the patient’s willingness and was confirmed by specific consent. Thereafter, patients were divided into two groups according to the number of TMZ cycles: Group A > 6 cycles and Group B ≤ 6 cycles. Patients were monitored to assess any events that might necessitate the discontinuation of treatment such as hematologic toxicity from TMZ on monthly blood tests. Disease progression was investigated on MRI every 3 months (evaluated by RANO criteria) [5]. In cases of suspected disease progression on MRI, an 11C-Methionine positron emission tomography/computed tomography (11C-MET PET/CT) was performed.

The study protocol was reviewed and approved by the appropriate Institutional Review Board, ensuring compliance with ethical standards.

Informed consent was obtained from all individual participants included in this study through dedicated forms. This process was conducted in accordance with the ethical standards of the institutional research committee and with the 1964 Helsinki declaration and its later amendments or comparable ethical standards.

Statistical analysis was conducted using SPSS v.26, focusing on descriptive statistics for the study population’s key characteristics and applying Student’s *t*-test and the Chi-squared test to compare groups. Survival rates were analyzed using the Kaplan–Meier method and log-rank test to compare curves, with the results expressed in medians and 95% Confidence Intervals (95%CIs). Univariate and multivariable Cox proportional hazard models were utilized to identify factors affecting survival, presenting the findings as hazard ratios (HRs) with 95%CIs. Additionally, a linear regression tree model in R was used to evaluate the impact of variables on Group A, considering *p*-values < 0.05 as statistically significant. Finally, missing data were treated using the multiple imputation strategy [6].

## 3. Results

### 3.1. Epidemiological Data

**Figure 1:**
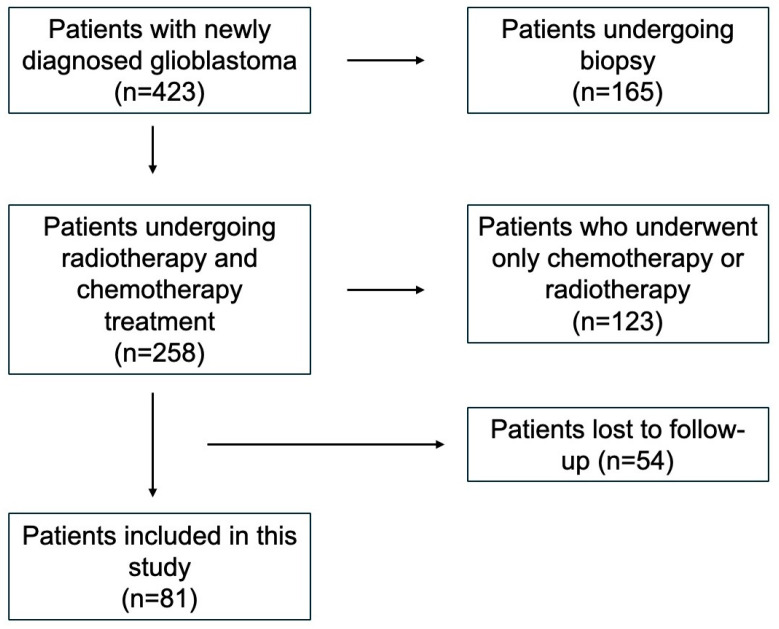
A schematic representation of the patient inclusion process.

From 2004 to 2018, 81 patients with newly diagnosed GB who met the inclusion criteria were enrolled (44 males—54.3%; 37 females—45.7%) (Figure 1).

The mean age at diagnosis was 60.6 ± 11.3 years (range 26–80 years). Group A (long-term TMZ) included 40 patients (24 males, 60%) with a mean age of 56.1 ± 11 years (range 26–75 years). Conversely, Group B (standard STUPP protocol) included 41 patients (20 males—48%) with a mean age of 64 ± 10.4 years (range 33–80 years). The age difference between Group A, who was younger, and Group B was statistically significant (*p* = 0.0014).

### 3.2. Radiological Evaluation: Tumor Volume and EOR

The mean preoperative tumor volume (i.e., the contrast-enhancing portion of the tumor) was 43.6 ± 20.6 cc (range 10–83.9 cc) in Group A and 46.5 ± 27.6 cc (range 5.1–147 cc) in Group B (*p* = 0.5942).

Postoperative residual tumor was present in 14 cases (3 in Group A and 11 in Group B); in all cases, it was near eloquent areas. The average EOR was over 99% in both groups (99.6% in Group A and 99.3% in Group B).

### 3.3. KPS Score

No statistically significant difference was observed between the mean preoperative (*p* = 0.124) and early postoperative (*p* = 0.793) KPS values between Groups A and B.

Furthermore, we noted a statistically significant improvement (*p* = 0.0044) in the mean KPS score at 6 months (85.5 ± 11.8; range 60–100) compared with the early postoperative score only in Group A (76.5 ± 15.7; range 40–100), while in Group B, the improvement (79 ± 18.3; range 50–100) was not significant (*p* = 0.395).

Six months after surgery, 34 patients (18 from Group B and 16 from Group A) exhibited unchanged clinical conditions, 33 were asymptomatic, and 14 (6 in Group A and 8 in Group B) reported nonspecific complaints.

### 3.4. MGMT Methylation and Ki-67 Analysis

MGMT promoter methylation status was significantly greater in Group A (39%) than in Group B (19%) (*p* = 0.003). In contrast, the levels of Ki-67 were significantly higher in Group B (30%) than in Group A (17.8%) (*p* = 0.010).

### 3.5. Assessment of OS and PFS

In Group A, the mean number of TMZ cycles was 24.1 ± 21 (range 7–101), and it was 3.6 ± 1.8 (range 1–6) in Group B. The mean OS was 35.9 ± 22.1 months (range 12–109) in Group A and 11.3 ± 7.3 months (range 3–33) in Group B. The mean PFS was 27.8 ± 22.9 months (range 4–103) in Group A and 7.5 ± 6.5 months (range 1–32) in Group B. Three patients in Group A died of non-neuro-oncological causes, never presenting with GB recurrence. Nine patients were still alive at the time of analysis, so the mean OS of the censored group was 44.8 months.

**Figure 2:**
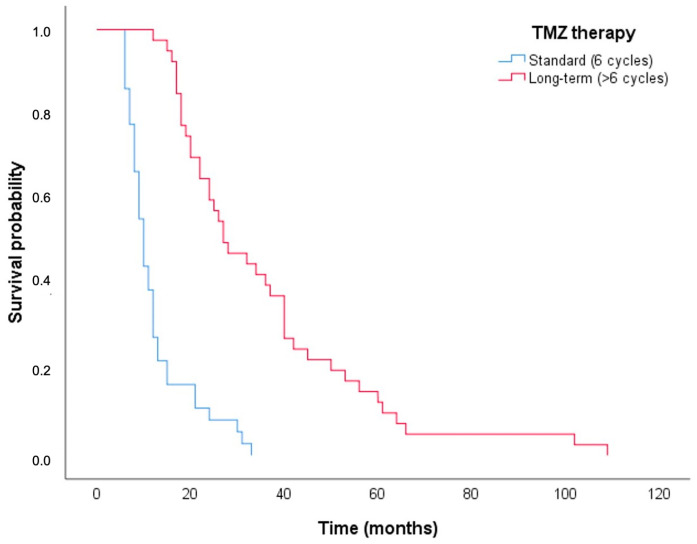
Graphical representation of OS according to Kaplan–Meier method. Group A in red (>6 TMZ cycles) and Group B in blue (6 TMZ cycles).

Group B included patients who received one to six cycles of TMZ therapy, although the complete STUPP protocol involves the administration of six cycles. This introduces a potential for biases that may impact the comparison of survival outcomes between groups. To mitigate this, our initial analysis focused on comparing OS exclusively among those in Group B who completed the recommended six cycles and those in the long-term TMZ group (>6 cycles of TMZ; Group A) using the Kaplan–Meier method (Figure 2).

**Figure 3:**
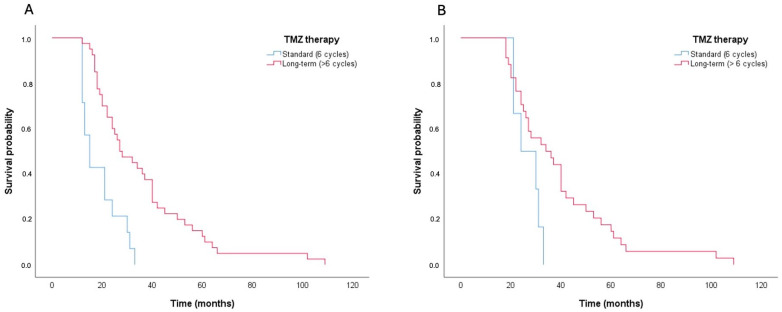
Graphical representation of OS according to Kaplan–Meier method. Group A in red (>6 TMZ cycles) and Group B in blue (6 TMZ cycles). (**A**) Survival analysis of patients with minimum survival of 12 months. (**B**) Survival analysis of patients with minimum survival of 18 months.

Specifically, patients in Group A demonstrated a prolonged OS compared to their counterparts in Group B (median = 27.0 months; 95%CI = 17.7–36.3, compared to median = 10.0 months; 95%CI = 8.5–11.5; *p* < 0.001, as determined by the log-rank test). Similar findings were observed in a focused analysis of patients who had achieved a minimum survival of 12 months (*n* = 54; 40 in Group A and 14 in Group B) or 18 months (*n* = 40; 36 in Group A and 4 in Group B) following the intervention (Figure 3).

Considering a minimum survival period of 12 months, patients in Group A demonstrated an extended OS compared to their counterparts in Group B (median = 27.0 months; 95%CI = 17.7–36.3, compared to median = 15.0 months; 95%CI = 11.4–18.6; *p* < 0.001). We observed a similar case in the analysis of patients with a minimum survival of 18 months (median = 34.0 months; 95%CI = 21.1–46.9 in Group A compared to median = 24.0 months; 95%CI = 13.2–34.8 in Group B; *p* = 0.044).

### 3.6. Predictors of OS

Since the two groups significantly differed in terms of age and MGMT methylation status, a univariate analysis was initially performed to assess the effects of these variables on OS. This analysis focused exclusively on patients from Group B who successfully underwent the recommended six cycles and those from Group A. The findings indicated that age, MGMT methylation status, and long-term TMZ therapy were potential predictors of OS (*p*-values < 0.01; Table 1). In the multivariable Cox regression model integrating these variables, long-term TMZ therapy emerged as an independent predictor of OS, significantly reducing the risk of death (HR = 0.15; 95%CI = 0.05–0.42; *p* < 0.001).

**Figure 4:**
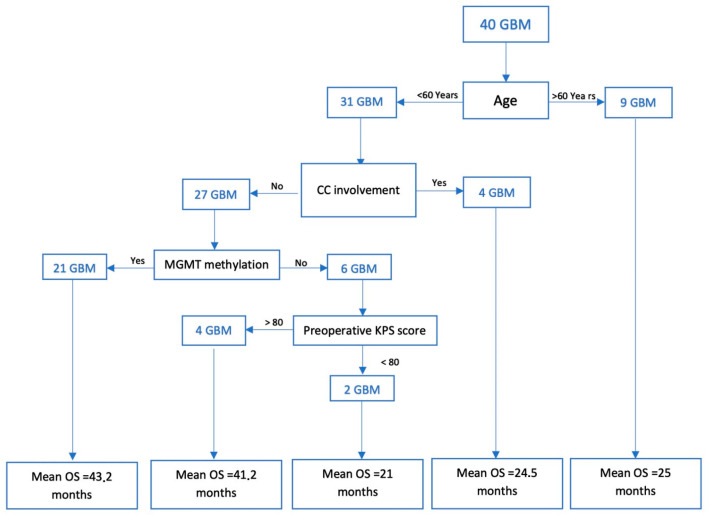
A graphical representation of the regression tree and the impact of individual variables on the operating system in patients in Group A.

To determine the impact of the selected clinical and radiological factors on outcome in Group A, a regression tree was structured in R. The parameters explored were age (greater or less than 60 years), corpus callosum invasion, EOR (GTR or STR), preoperative KPS score (greater or less than 80), and MGMT methylation status. The results are summarized in Figure 4. It would appear that TMZ treatment extension is more effective in improving outcomes in younger patients without corpus callosum invasion, with MGMT promoter methylation, and treated with GTR.

We also explored data on relapsing GB during follow-up. Overall, relapsing GB was reported 23% (19/81) and specifically 30% (12/40) in Group A and 17% (7/41) in Group B. In reoperated patients, PFS was significantly (*p* = 0.013) better in Group A (23.3 months, range 10–48) than in Group B (8.4 months, range 4–19).

### 3.7. Complications

Comprehensively, chemotherapy-related complications were reported in 18.5% (15/81) of patients. There was no significant difference (*p* = 0.1149) in the complication rate between Group A (20%, 8/40) and Group B (17%, 7/41). Specifically, in Group A, there were four grade I complications (thrombocytopenia, creatinine elevation, and hypertransaminasemia), two grade II (asthenia and hypertransaminasemia), and two grade III (thrombocytopenia and asthenia). In Group B, there were two grade I events, three grade II events, one grade III event, and one grade IV event (severe thrombocytopenia). However, these complications led to the interruption of TMZ treatment in only one patient in Group B.

## 4. Discussion

The duration of adjuvant chemotherapy with TMZ was originally established arbitrarily, without any comparative studies conducted. In clinical practice, the number of cycles is often variable, and patients commonly receive up to 12 or more TMZ cycles [7].

Although several studies investigated the efficacy of long-term adjuvant TMZ therapy, no univocal data are currently available, and this topic remains controversial. In 2014, we reported the longest experience of TMZ administration (up to 101 cycles) in newly diagnosed GB [8]. Other authors have reported findings supporting the efficacy of the extended STUPP protocol beyond six cycles with an acceptable safety profile [9,10,11]. Nevertheless, these studies often analyze data on all types of high-grade gliomas, not focusing exclusively on GB, probably because of the difficulty in conforming histologic diagnoses to the new 2021 WHO classification, lacking the required molecular data.

In 2022, Wang et al. compared the efficacy of the standard adjuvant TMZ protocol with the extended treatment (up to 12 cycles) in a retrospective, multicenter cohort study. In patients with GB, PFS and OS were significantly poorer in the standard STUPP group than in the extended STUPP group (PFS: 12 vs. 20 months, *p* = 0.001; OS: 13 vs. 36 months, *p* < 0.001). They observed a higher toxicity of TMZ in extended treatment patients with the most common adverse reaction being nausea/vomiting [10].

In 2021, Huang et al. performed a single-center, retrospective study of GB patients based on the 2007 WHO criteria for diagnosis. They found that long-term adjuvant TMZ chemotherapy improved PFS (15 vs. 20.1 months, *p* = 0.008) but failed to significantly improve OS (19.4 vs. 25.6 months, *p* = 0.152) [12]. Analogous studies report a similar result [13,14].

Some authors discourage long-term TMZ as data analysis failed to demonstrate a survival advantage. Among them, those of a 2020 Spanish multicenter, randomized, phase II study (GEINO 14-01) found no significant differences in PFS and OS between two groups. Furthermore, patients who underwent the extended protocol exhibited a higher rate of toxicity with lymphopenia, thrombocytopenia, and nausea/vomiting (*p* = 0.001) [15].

In 2024, a combined analysis examined data from two prospective randomized studies on twelve-month extended TMZ treatment. The key findings include the lack of significant improvement in 6-month PFS and OS with extended TMZ treatment. Despite its extensive scope, the study revealed several limitations, such as the absence of GB patient classification according to the latest WHO 2021 criteria, the inclusion of biopsy in the EOR criteria, and the inability to identify the subgroups of patients who might derive greater benefit from extended TMZ treatment [2].

Gupta et al. (2022) conducted an updated systematic review and meta-analysis including 358 patients from four prospective randomized controlled trials comparing standard (6 cycles) versus extended (>6 cycles) adjuvant TMZ in newly diagnosed GB [16]. The primary endpoint was OS, while PFS and grade ≥3 hematologic toxicity were secondary outcomes. The pooled results showed no statistically significant improvement in survival with extended TMZ. Specifically, the hazard ratio for progression was 0.82 (95% CI: 0.61–1.10; *p* = 0.18) and for death 0.87 (95% CI: 0.60–1.27; *p* = 0.48). Extended TMZ also did not significantly increase hematologic toxicity (RR = 2.01; 95% CI: 0.83–4.87; *p* = 0.12). Despite including only randomized controlled trials, the certainty of evidence was graded as low, mainly due to small sample sizes, open-label design, and the lack of MGMT-based stratification in most studies. Furthermore, no meaningful subgroup analysis could be performed based on MGMT methylation due to insufficient data.

Anvari et al. (2024) conducted a randomized, single-blind, two-arm controlled trial involving 100 patients with newly diagnosed high-grade gliomas (GB and anaplastic astrocytoma) to compare 12 versus 6 cycles of adjuvant TMZ [17]. At a median follow-up of 26 months, the median OS was 35 months in the 6-cycle group and 23 months in the 12-cycle group (*p* = 0.19). Similarly, the median PFS was 18 months for 6 cycles and 16 months for 12 cycles (*p* = 0.88). No significant survival benefit was observed with the extended regimen, and the safety profile remained comparable between groups, with only mild adverse events more frequently reported in the 12-cycle group. However, several limitations must be acknowledged. Firstly, patients were classified according to the WHO 2016 CNS tumor classification, without an assessment of IDH mutation and MGMT promoter methylation, including only patients with KPS > 60%. Moreover, biopsy-only procedures were considered as part of the extent of resection.

In our study, the long-term TMZ group had improved PFS and OS (PFS: 27.8 vs. 7.5 months, *p* = 0.00001; OS: 35.9 vs. 11.3 months, *p* = 0.0001).

We acknowledge that Group B patients received fewer TMZ cycles than the full STUPP protocol, which calls for six cycles, potentially skewing survival outcome comparisons. Addressing this, the initial comparison of OS was conducted between Group B patients who completed six cycles and those in Group A (who received more than six cycles), using Kaplan–Meier analysis. This revealed that Group A had a significantly longer median OS of 27.0 months compared to Group B’s median of 10.0 months.

Upon examining patients who lived at least 12 months, Group A patients experienced longer OS than those in Group B (27.0 months versus 15.0 months, *p* < 0.001). Similarly, in the subset of patients reaching at least 18 months post-treatment, Group A showed a median OS of 34.0 months compared to 24.0 months for Group B, with statistical significance (*p* = 0.044). Regarding prognostic factors, the methylation status of the MGMT gene and extended treatment with TMZ were significant indicators of OS. Further analysis using a multivariable Cox regression model identified prolonged TMZ treatment as an independent factor that significantly decreased mortality risk (hazard ratio = 0.15, *p*-value < 0.001).

Finally, our study also demonstrated the safety of prolonged treatment with TMZ. Indeed, we did not notice a significant difference in the rate of side effects between the two groups, and these led to treatment discontinuation in only one case in Group B.

While acknowledging the potential trade-offs of extended TMZ administration, it is worth noting that the overall cost remains relatively acceptable, especially considering that TMZ is an oral agent administered only a few days per month. This regimen is generally well tolerated and manageable, both clinically and psychologically. Nevertheless, the possibility of cumulative toxicity should not be overlooked. On balance, if survival advantages are demonstrated, the benefits of prolonging treatment may outweigh the associated burdens.

### Limitations

We acknowledge that the small sample size is a primary limitation of our study, yet we analyzed a homogeneous group of patients, yielding clinically useful and statistically significant results. The main constraints stem from the observational design of this study, potentially introducing biases, especially selection and survival biases. We attempted to mitigate these biases by excluding non-adherent patients to the standard protocol and using multivariable models for analysis adjustment. However, the survival bias remains a challenge, as it could distort the perceived benefits of a treatment. We tried to control this by conducting subgroup analyses for patients surviving at least 12 or 18 months. Future prospective, randomized studies are necessary to confirm our findings. Furthermore, the two groups being compared may differ in factors other than the type of treatment (in our case, specifically, they differed in age and MGMT methylation status). To mitigate the potential effects of these differences, a multivariable regression model was applied, which included group assignment and other factors. However, it cannot be ruled out that other differences, not identified in the comparison—possibly due to the small sample size—or not considered, may have at least partially influenced the results.

## 5. Conclusions

Our analysis demonstrates PFS and OS advantages in the extended STUPP cohort and suggests that young patients without corpus callosum invasion, with MGMT promoter methylation, and treated with GTR are the best candidates for long-term TMZ chemotherapy. In addition, no significant safety difference emerged between extended and standard TMZ treatment.

## Figures and Tables

**Table 1:** Univariate and multivariable analyses of predictors influencing overall survival according to Cox model.

Characteristics	Univariate Model		Multivariable Model ^a^	
	HR (95%CI)	*p*-Value	HR (95%CI)	*p*-Value
Age ^b^	1.03 (1.01–1.06)	0.014	1.02 (0.99–1.05)	0.276
MGMT methylation (yes vs. no)	0.43 (0.24–0.76)	0.004	1.08 (0.44–2.67)	0.868
TMZ protocol (long-term vs. standard)	0.17 (0.10–0.29)	<0.001	0.15 (0.05–0.42)	<0.001

^a^ The multivariable model incorporates independent variables that demonstrated associations with OS in the univariate analyses. ^b^ The results are presented as HRs along with their corresponding 95%CIs for each one-unit increase in the independent variable.

## Data Availability

The raw data supporting the conclusions of this article will be made available by the authors on request.

## Abbreviations

The following abbreviations are used in this manuscript:

5-ALA	5-aminolevulinic acid
11C-MET PET/CT	11C-Methionine positron emission tomography/computed tomography
CNS	central nervous system
CTCAE	Common Terminology Criteria for Adverse Events
EOR	extent of resection
GB	glioblastoma
GTR	gross total resection
iCT	intraoperative computed tomography
IONM	intraoperative neurophysiological monitoring
KPS	Karnofsky Performance Status
MGMT	methylguanine-DNA methyltransferase
MRI	magnetic resonance imaging
OS	overall survival
PFS	progression-free survival
STR	subtotal resection
TMZ	temozolomide
WHO	World Health Organization

## References

[1] Stupp R., Mason W.P., van den Bent M.J., Weller M., Fisher B., Taphoorn M.J.B., Belanger K., Brandes A.A., Marosi C., Bogdahn U. (2005). Radiotherapy plus concomitant and adjuvant temozolomide for glioblastoma. N. Engl. J. Med..

[2] Gately L., Mesía C., Sepúlveda J.M., Del Barco S., Pineda E., Gironés R., Fuster J., Hong W., Dumas M., Gill S. (2024). A combined analysis of two prospective randomised studies exploring the impact of extended post-radiation temozolomide on survival outcomes in newly diagnosed glioblastoma. J. Neurooncol..

[3] Karschnia P., Young J.S., Dono A., Häni L., Sciortino T., Bruno F., Juenger S.T., Teske N., Morshed R.A., Haddad A.F. (2023). Prognostic validation of a new classification system for extent of resection in glioblastoma: A report of the RANO resect group. Neuro-Oncology.

[4] Basch E., Becker C., Rogak L.J., Schrag D., Reeve B.B., Spears P., Smith M.L., Gounder M.M., Mahoney M.R., Schwartz G.K. (2021). Composite grading algorithm for the National Cancer Institute’s Patient-Reported Outcomes version of the Common Terminology Criteria for Adverse Events (PRO-CTCAE). Clin. Trials.

[5] Wen P.Y., Macdonald D.R., Reardon D.A., Cloughesy T.F., Sorensen A.G., Galanis E., DeGroot J., Wick W., Gilbert M.R., Lassman A.B. (2010). Updated response assessment criteria for high-grade gliomas: Response assessment in neuro-oncology working group. J. Clin. Oncol..

[6] Sterne J.A., White I.R., Carlin J.B., Spratt M., Royston P., Kenward M.G., Wood A.M., Carpenter J.R. (2009). Multiple imputation for missing data in epidemiological and clinical research: Potential and pitfalls. BMJ.

[7] Blumenthal D.T., Gorlia T., Gilbert M.R., Kim M.M., Nabors L.B., Mason W.P., Hegi M.E., Zhang P., Golfinopoulos V., Perry J.R. (2017). Is more better? The impact of extended adjuvant temozolomide in newly diagnosed glioblastoma: A secondary analysis of EORTC and NRG Oncology/RTOG. Neuro-Oncology.

[8] Barbagallo G.M., Paratore S., Caltabiano R., Palmucci S., Parra H.S., Privitera G., Motta F., Lanzafame S., Scaglione G., Longo A. (2014). Long-term therapy with temozolomide is a feasible option for newly diagnosed glioblastoma: A single-institution experience with as many as 101 temozolomide cycles. Neurosurg. Focus.

[9] Darlix A., Baumann C., Lorgis V., Ghiringhelli F., Blonski M., Chauffert B., Zouaoui S., Pinelli C., Rech F., Beauchesne P. (2013). Prolonged administration of adjuvant temozolomide improves survival in adult patients with glioblastoma. Anticancer Res..

[10] Seiz M., Krafft U., Freyschlag C.F., Weiss C., Schmieder K., Lohr F., Wenz F., Thomé C., Tuettenberg J. (2010). Long-term adjuvant administration of temozolomide in patients with glioblastoma multiforme: Experience of a single institution. J. Cancer Res. Clin. Oncol..

[11] Wang J., Huang Y., Zhao F., Chen J., He L., Liu Z., Pei Y., Wei Z., Li R., Ai P. (2022). Standard or extended STUPP? Optimal duration of temozolomide for patients with high-grade gliomas: A retrospective analysis. J. Neuro-Oncology.

[12] Huang B., Yu Z., Liang R. (2021). Effect of long-term adjuvant temozolomide chemotherapy on primary glioblastoma patient survival. BMC Neurol..

[13] Gramatzki D., Kickingereder P., Hentschel B., Felsberg J., Herrlinger U., Schackert G., Tonn J.-C., Westphal M., Sabel M., Schlegel U. (2017). Limited role for extended maintenance temozolomide for newly diagnosed glioblastoma. Neurology.

[14] Skardelly M., Dangel E., Gohde J., Noell S., Behling F., Lepski G., Borchers C., Koch M., Schittenhelm J., Bisdas S. (2017). Prolonged Temozolomide Maintenance Therapy in Newly Diagnosed Glioblastoma. Oncologist.

[15] Balana C., Vaz M.A., Manuel Sepúlveda J., Mesia C., del Barco S., Pineda E., Muñoz-Langa J., Estival A., Peñas R.d.L., Fuster J. (2020). A phase II randomized, multicenter, open-label trial of continuing adjuvant temozolomide beyond 6 cycles in patients with glioblastoma (GEINO 14-01). Neuro-Oncology.

[16] Gupta T., Talukdar R., Kannan S., Dasgupta A., Chatterjee A., Patil V. (2022). Efficacy and safety of extended adjuvant temozolomide compared to standard adjuvant temozolomide in glioblastoma: Updated systematic review and meta-analysis. Neuro-Oncology Pract..

[17] Anvari K., Seilanian Toussi M., Saghafi M., Javadinia S.A., Saghafi H., Welsh J.S. (2024). Extended dosing (12 cycles) vs conventional dosing (6 cycles) of adjuvant temozolomide in adults with newly diagnosed high-grade gliomas: A randomized, single-blind, two-arm, parallel-group controlled trial. Front. Oncol..

